# Extremely Elevated Prostate-Specific Antigen in Acute Prostatitis: A Case Report

**DOI:** 10.7759/cureus.43730

**Published:** 2023-08-18

**Authors:** Anamika Nepal, Prabhat Sharma, Shristi Bhattarai, Zubin Mahajan, Akhya Sharma, Ashok Sapkota, Aviskar Sharma

**Affiliations:** 1 Internal Medicine, Shankarapur Hospital, Kathmandu, NPL; 2 Internal Medicine, John H. Stroger, Jr. Hospital of Cook County, Chicago, USA; 3 Internal Medicine, MacNeal Hospital, Chicago, USA; 4 Internal Medicine, MacNeal Hospital, Berwyn, USA; 5 Neurosciences, Tata Main Hospital, Jamshedpur, IND; 6 Medicine, Kasturba Medical College, Manipal, IND

**Keywords:** urinary tract infection (uti), prostate cancer (pca), prostate-specific antigen (psa), acute prostatitis, chronic prostatitis

## Abstract

Elevated prostate-specific antigen (PSA) levels are mostly suggestive of prostate cancer, but they are elevated in non-cancerous prostatic conditions as well. However, extreme levels of PSA as reported here have not been observed in cases other than prostatic cancer so far. Our patient had a significantly elevated PSA of 1,398 ng/mL in acute prostatitis. The purpose of this case report is to review the patient's atypical and rare presentation of extremely high PSA in acute prostatitis in the background of benign prostatic hyperplasia (BPH) and chronic prostatitis.

## Introduction

Elevated prostate-specific antigen (PSA) levels are concerning for underlying prostate cancer, although there are non-cancerous prostatic conditions that can result in elevated PSA levels. Urinary tract infection (UTI), benign prostatic hyperplasia (BPH), and prostatitis are some conditions that result in elevated PSA levels [1]. No correlation of low PSA levels to prostatic cancer has been established, but extremely high levels of PSA are almost always suggestive of prostate cancer [2]. Here, we present a case of an elderly patient with a severely enlarged prostate and extremely elevated PSA, whose prostatic biopsy revealed acute and chronic inflammation of the prostate and did not reveal prostatic cancer.

## Case presentation

An 81-year-old male was admitted to our hospital with worsening mental status. He was a nursing home resident with multiple comorbidities including Alzheimer’s dementia, congestive heart failure, hypertension, BPH, and a recurrent history of UTIs. On examination, he was tachycardic, hypotensive, and hypoxic. He had a tense lower abdomen concerning bladder distension. The digital rectal exam revealed an enlarged and boggy prostate. His bladder scan revealed 700 mL of urine, drained by a urinary catheter. His labs were significant for leukocytosis, acute kidney injury, and pyuria. He was admitted with a diagnosis of acute septic encephalopathy secondary to UTI and acute hypoxic respiratory failure secondary to community-acquired pneumonia. His urine culture grew Escherichia coli which was resistant to fluoroquinolones and trimethoprim-sulphamethoxazole. He was treated in the intensive care unit, initially with a broad-spectrum antibiotic (meropenem), which was later deescalated to ampicillin based on culture and sensitivity results, oxygen supplementation, and non-invasive ventilation.

**Figure 1:**
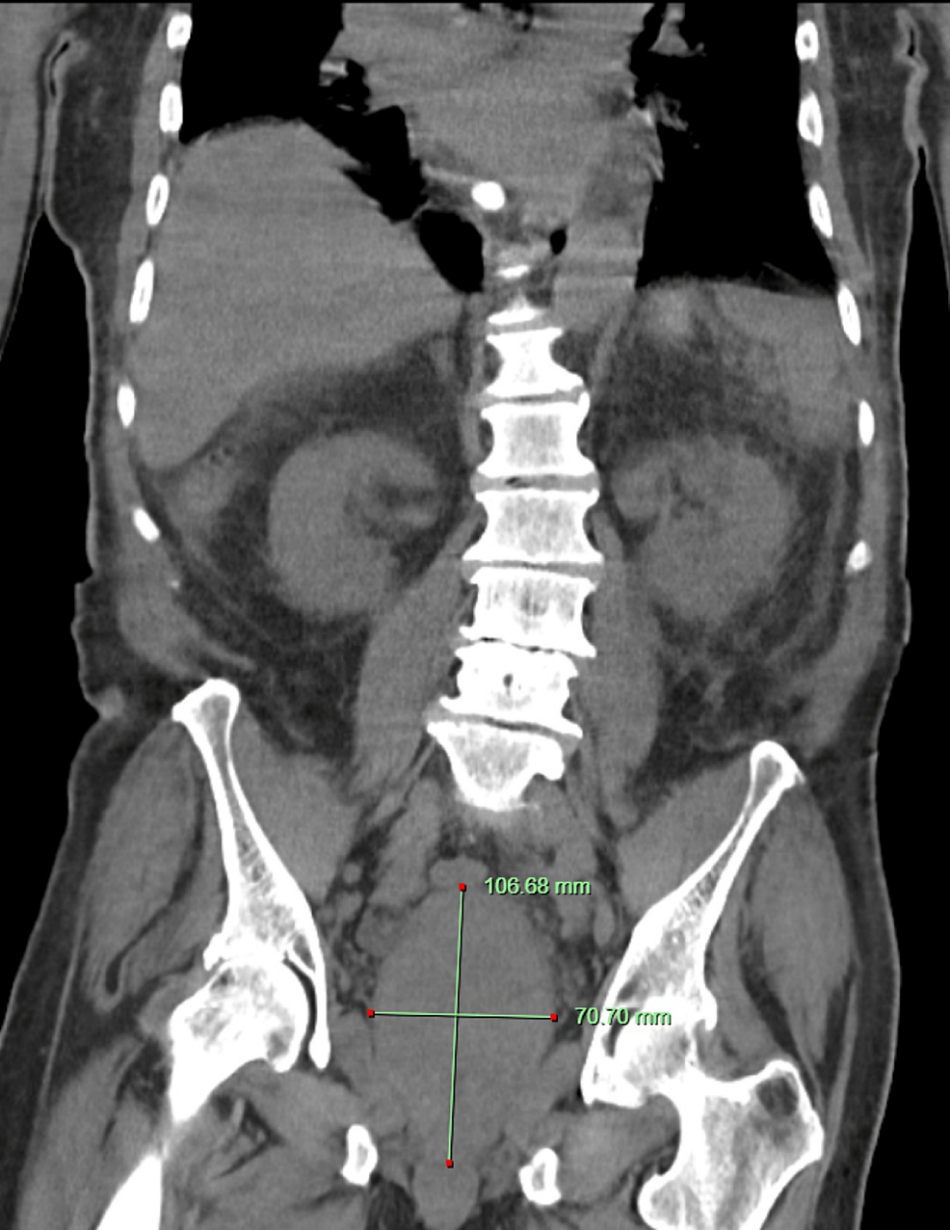
CT scan of coronal view of abdomen and pelvis without intravenous contrast showing enlarged prostate gland. CT: Computed Tomography

**Figure 2:**
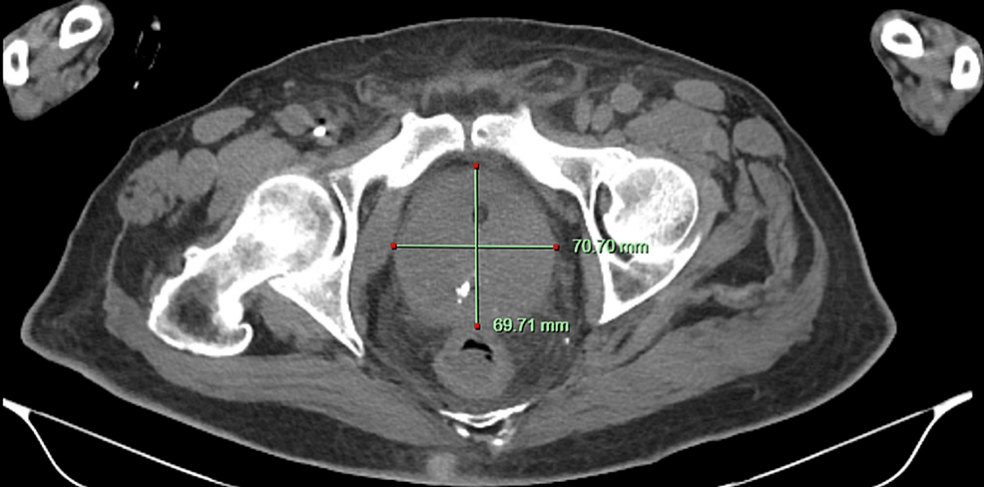
CT scan of sagittal section of the pelvis showing enlarged prostatic gland. CT: Computed Tomography

Computed tomography (CT) scan of the abdomen and pelvis without contrast, which was done as a part of the workup revealed a severely enlarged prostate (Figures 1, 2). The prostatic size was radiologically calculated as 275 cc. His total PSA levels were elevated to 1,398 ng/mL. We consulted the urology team because of high suspicion of prostatic cancer, who recommended trans-rectal ultrasound (TRUS) guided prostate biopsy. A total of 15 core-needle biopsy fragments of the prostate gland were obtained for pathologic analysis. He improved clinically and was discharged to the nursing home on ertapenem on day 5 of admission. He was advised to return for a follow-up in two weeks to discuss the biopsy results.

The prostate biopsy revealed benign prostatic tissue with acute and chronic inflammation and focal necrosis. No malignant cells were seen, and the gram stain was negative for any organisms. Whole-body bone scan using Technetium-99m-methylene diphosphonate (Tc-99m-MDP) was negative for metastatic bone lesions. He was lost to follow-up at our hospital, so we do not have the repeat PSA measured after discharge. The patient’s power of attorney provided consent for this case report.

## Discussion

The normal male serum PSA levels are usually less than 4 ng/mL [3]. PSA level is widely used as a screening tool for prostate cancer, but elevated levels of PSA are often seen in non-cancerous prostatic conditions. Some examples are benign prostatic hyperplasia, acute prostatitis, chronic prostatitis/chronic pelvic pain syndrome, prostate abscess, urinary tract infection in males, and granulomatous prostatitis among others [1,4]. We believe that the cause of PSA elevation in our patient was most likely acute prostatitis in the background of benign prostatic hyperplasia and chronic prostatitis. Digital stimulation of the prostate gland during rectal examination also contributed to the increase in PSA level.

There are case reports reporting non-cancerous causes of high PSAs, but they have never been shown to be more than 100 ng/mL. Generally, they are seen at a PSA level of less than 50 ng/mL [2]. A large, benign prostatic cyst was reported to be associated with a PSA level of 79 ng/mL [5]. Also, high levels of PSA were found in patients with cardiogenic shock without any prostate disease as reported by Fernandez-Galan et al. [6]. The highest PSA levels ever recorded is 23,162 ng/mL in prostate cancer [2]. Our patient had a PSA elevation of 1,398 ng/mL, a level that almost always suggests an underlying diagnosis of prostate cancer. Extremely high PSA level also implies a high tumor burden [2]. From the use of the equation developed by Kato et al., the predicted tumor volume would have been 425 cc if our patient actually had prostate cancer. But the equation was only used to estimate tumor volume in those who were already diagnosed with prostate cancer [7].

A cross-sectional study done on 72 patients with prostatitis revealed that PSA level was elevated above 4 ng/mL in 71% with acute prostatitis, 15% with chronic bacterial prostatitis, and 6% with abacterial prostatitis [8]. In febrile UTIs in males, the prostate and seminal vesicles are frequently affected, where PSA may be a helpful indicator of prostatic infection, although current guidelines usually do not recommend testing PSA in all patients with acute bacterial prostatitis [9]. Urine culture is often the first step in diagnosing the condition. E. coli remains the most common organism responsible for it, and the first line treatment is fluoroquinolone [9]. Our patient was resistant to fluoroquinolones, so the initial antibiotic meropenem was de-escalated to ampicillin and later ertapenem. In patients who have active bacterial prostatitis with elevated PSA, the PSA levels are usually repeated after the resolution of acute infection to see if it returns to baseline. If the PSA levels remain elevated, further diagnostic procedures for prostate cancer are done (i.e., prostate biopsy). However, it has also been shown that some patients' PSA levels slowly decreased, which suggested a lengthy healing phase and it should be considered when considering prostate cancer [10].

However, in our case, we had a high pre-test probability of prostate cancer given the enlarged prostate gland and PSA levels. So, a prostate biopsy was done during the admission. We did not get an opportunity to follow up with repeat PSA levels after treatment, which is a limitation of our report. We could not observe whether the PSA levels improved, persisted, or worsened after the treatment.

## Conclusions

Significantly elevated PSA levels > 1,000 ng/mL can be observed in non-cancerous prostatic conditions such as acute prostatitis among others. So, whenever such high PSA levels are encountered, it is important to explore other possible causes. Obtaining PSA levels in acute bacterial prostatitis can be avoided as it can result in very high levels of PSA, which can create a dilemma in management. Acute bacterial prostatitis should be initially treated with appropriate antibiotics and repeat PSA levels should be obtained after the acute condition has resolved. The decision to perform a prostate biopsy immediately might be reconsidered in such cases.
